# Fostering self-efficacy in Chinese university athletes: the mediating roles of psychological resilience and emotional regulation and the moderating role of autonomy-supportive coaching

**DOI:** 10.3389/fpsyg.2025.1664339

**Published:** 2025-12-15

**Authors:** Xiao Zhang, Safeer Ullah Khan

**Affiliations:** 1 Department of Public and Ideological and Political Courses, Hubei Sports Vocational College, Wuhan, China; 2 Shenzhen University, Shenzhen, China

**Keywords:** autonomy-supportive coaching, China, mental toughness, PLS-SEM, self-efficacy

## Abstract

This study explores how mental toughness contributes to the psychological sustainability of athletes by enhancing emotional regulation and psychological resilience, ultimately fostering self-efficacy, particularly in environments supported by autonomy-supportive coaching. The study examines mental toughness (MT), a dispositional resource for goal-focused persistence under pressure, and its effects on performance self-efficacy (PSE) through the mediating roles of emotional regulation (ER) and psychological resilience (PR), with autonomy-supportive coaching (ASC) as a contextual moderator. University athletes in China face intense dual academic-athletic demands within a collectivist culture that may suppress autonomy and undermine self-efficacy, yet little research addresses how psychological and coaching factors jointly shape their confidence. Using purposive sampling, we recruited 581 active university athletes across China who met the eligibility criteria: (1) currently enrolled as a university student, and (2) actively participating in organized sports for at least one year. Participants completed validated scales measuring the target constructs. We analyzed the data through structural equation modeling (SEM) in Smart PLS. Results reveal that MT positively affects ER and PR, which in turn significantly enhance PSE. Both ER and PR mediate the relationship between MT and PSE, while ASC strengthens the positive effects of MT on ER and PR. Theoretically, the study highlights the role of internal psychological resources and supportive environments in sustaining athlete development over time, offering a framework that bridges sport psychology with broader concepts of personal and developmental sustainability. The study provides practical guidance for coaches aiming to foster mental toughness and psychological skills to improve athletes' confidence and psychological resilience.

## 1 Introduction

Self-efficacy is a crucial concept in the area of sporting performance, as it can both influence how an athlete approaches challenges, sustain motivational levels, and execute actions when under pressure (Tokarska and Rogowska, 2025). Bandura (2023) has defined self-efficacy as the individual's perception of the capacity to plan and execute the actions which are necessary to reach a particular goal. In this context, self-efficacy influences the athlete's confidence to remain involved in training, to adapt in the competition and to recover from failures (Anstiss et al., 2020). Research has suggested that increased self-efficacy is related to greater performance, psychological resilience, and goal achievement across a range of sports (Aizava et al., 2024). To understand its role in sport success, researchers and practitioners associated with sport psychology should study the psychological and contextual factors that support the development of self-efficacy.

Prior studies have identified a number of factors that shape the experience of self-efficacy development in athletes. There are two sources with regard to self-efficacy development that are most likely well-accepted. Previous performance accomplishments are a source that serves as an objective indicator of ability. Verbal persuasion may help build self-efficacy when encouragement is given by others, such as coaches or teammates (Feltz and Lirgg, 2001). Vicarious experiences, namely seeing others who are similar to you perform, also encourage an athlete's self-belief in their own capabilities, especially in team sports (Zagórska and Guszkowska, 2014). Lastly, an athlete's individual emotional and physiological states can provide considerable influence on their self-efficacy development in high-pressure situations. Factors such as the regulation of anxiety, appraisal of stress, and control of arousal can shape an athlete's confidence in these situations (van Rens et al., 2021). The above mechanisms underpinning self-efficacy are well researched, plus we have an understanding how these mechanisms combine. However, it is becoming evident that in addition to the above constructs, there may be a number of individual psychological traits that may influence an athlete's self-efficacy development. One such trait, mental toughness, is fascinating, yet largely unexplored, and while there are theoretical overlaps with psychological resilience, confidence and emotional control, there seems to be little empirical work examining how mental toughness, as a precursor, influences the psychological mechanisms, such as resilience and emotional regulation, which shape self-efficacy. Although mental toughness (MT) conceptually overlaps with psychological resilience and grit, theoretically and empirically it is distinct from both. While grit involves persistent and obstinate directedness toward some long-term goal (Duckworth et al., 2007), MT is concerned with flexibility and adaptability amidst competitive and high-pressure goal-directed performance (Gucciardi, 2017). Similarly, while psychological resilience implies rebounding in the face of adversity, MT implies a confrontation of stress with an orientation to the furtherance of goal-directedness (Cowden et al., 2016; Gucciardi, 2017). Emotional regulation, on the other hand, is a distinctive self-regulation process that MT may facilitate but does not entail. The differentiation of these cognitions is important because it helps contribute to the theoretical exactitude, as well as the operationalization, of the recursively conceptualized dispositional antecedent of MT that facilitates but does not explicitly constitute downstream regulation such as psychological resilience and emotional regulation. This discussion of mental toughness raises an important line of inquiry and could present mental toughness as a core psychological resource in the self-efficacy development process.

Mental toughness has been recognized as an important psychological trait that helps an athlete deal with stress, adversity, and high-pressure situations (Çetin et al., 2025). It includes characteristics such as perseverance, confidence, emotional control, and the ability to remain focused in the face of adversity (Tokarska and Rogowska, 2025). One of the main avenues mental toughness might influence performance-related outcomes is through psychological resilience–the ability to bounce back quickly from setbacks while functioning well under pressure (Bandura, 2023). Mentally tough athletes will frequently adaptively meet adversity, remaining motivated in the face of setbacks and viewing adversity as an opportunity for growth (Anstiss et al., 2020). Psychological resilience has been positively associated with greater self-efficacy since athletes who are able to bounce back quickly from setbacks who are able to persist through obstacles tend to develop a higher belief in their capabilities (Aizava et al., 2024). Therefore, it is reasonable to expect that mental toughness facilitates psychological resilience, which subsequently enhances self-efficacy. It is critical to note that psychological resilience defined as the capacity to adapt positively in the face of significant adversity (Fletcher and Sarkar, 2016), is not inherent to mental toughness but rather a malleable outcome it may foster. MT provides the cognitive-affective foundation (e.g., confidence, control) that enables resilient responses, but the two constructs operate at different levels: MT as a relatively stable trait-like resource, and psychological resilience as a dynamic process of adaptation.

Another vital mechanism by which mental toughness is related to self-efficacy is emotional regulation which is defined as the ability to manage and manipulate the emotional response in a high-stress situation (Feltz and Lirgg, 2001). Mentally tough athletes generally have better emotional control, enabling them to remain composed, focused, and goal-directed whilst dealing with competitive stress, poor performance, or external distractions (Zagórska and Guszkowska, 2014). This emotional steadiness allows the mentally tough athlete to lessen performance anxiety, prevent negative emotional spirals, and keep them psychologically ready (van Rens et al., 2021), which all facilitate high self-efficacy and eventually self-regulation (Çetin et al., 2025). When athletes can regulate their emotions effectively, they are more likely to be able to interpret hard situations positively and view themselves as having the capability to achieve an outcome (Mendizabal, 2024). Hence, mental toughness could enhance self-efficacy through the development of emotional regulation as one of the key psychological skills of managing the stress of competitive demands.

Although individual psychological traits such as mental toughness are critical in defining athletic adaptive capacity, the social environment, and in particular the type of coaching style employed, also plays a major role in determining the way in which these traits are expressed and developed. In particular, the autonomy-supportive coaching which arises from Self-Determination Theory (Deci and Ryan, 2012) is construed to mean a coaching style which gives an opportunity for the athlete to make choices, facilitates self-directed behavior and takes the athlete's opinions and feelings into account (Amorose and Anderson-Butcher, 2015). This type of climate fosters intrinsic motivation and encourages psychological skills through the development of autonomy, competence and relatedness (Noble et al., 2016). Athletes who are involved in work with autonomy-supportive coaches may feel to be psychologically motivated and empowered to face the demands of performance (Burrell, 2016). This type of climate may have an effect on the expression of internal traits such as mental toughness, which causes athletes to be able to become more fully engaged in experiences which foster psychological resilience and stimulate self-regulation with regard to emotional feelings. However, despite the increased interest in MT and the interactions of coaches, the number of empirical studies which have examined the moderating effect of an autonomy-supportive context on the relationship between MT and important psychological mechanisms (e.g., emotion regulation, psychological resilience) is scant, particularly in non-Western settings. University athletes in China represent a compelling population: they navigate intense academic-athletic dual demands within a collectivist culture that often emphasizes obedience over autonomy (Song et al., 2024; Zhou and Zhang, 2024). This context may enhance the protection emanating from autonomy-supportive coaching, which may render it a crucial moderator in the MT-self-efficacy relation. Thus, it is reasonable to suggest that the relationship between mental toughness and psychological resilience, as well as the relationship between mental toughness and emotion regulation, will be stronger in an autonomy-supportive coaching environment. This moderating effect illustrates the vital interaction between individual attributes and the social context which influences athlete development. Therefore, the following research questions are posited based on the aforementioned insights:

**RQ1**: Does mental toughness positively influence psychological resilience and emotional regulation among athletes?
**RQ2:** Do psychological resilience and emotional regulation mediate between mental toughness and athletes' performance self-efficacy?
**RQ3:** Does autonomy-supportive coaching moderate the relationship between mental toughness and both psychological resilience and emotional regulation?

This research provides several significant contributions to the field of sport psychology and athlete sustainable development. First, this research furthers the current literature on the antecedents of self-efficacy. By considering mental toughness as a distinct internal resource of performance that can act to indirectly shape self-beliefs through psychological resilience and emotional regulation, this research posits an integrated model for the use of mental toughness in sport. Although prior studies in the area of mental toughness have studied these proposed variables individually, the integrated use of the psychological protective processes associated with mental toughness posits a holistic view of the psychological processes that underlie self-efficacy. The inclusion of autonomy-supportive coaching as a moderator adds depth to our understanding of how supportive environments contribute to the sustainability of self-efficacy development in athletes over time. By studying both internal traits, contextual or external environments, this integrated model of mental toughness unites dispositional and contextual purposes that can better inform our understanding of athlete functioning. Finally, the model will also have implications for coaches, sport psychologists, or training programs interested in ways to enhance athlete confidence and performance through improved individual attributes and supportive psychological context to the development of mental toughness.

## 2 Literature review

MT is an important construct within sport psychology, influenced by its widely perceived importance as a psychological resource that allows athletes to consistently perform at high levels when under pressure, adversity, and stress. Gucciardi (Gucciardi, 2017) defines MT as a state-like psychological resource characterized by intentional, flexible, and effective capacity during goal-related and task-oriented endeavors, especially in adverse situations. While MT is often viewed as related to or as a fixed trait, MT is viewed as more context sensitive and developable over time, which allows individuals to flexibly adapt their actions based on the demands of a situation (Gucciardi, 2017). While early definitions of MT tended to focus on a framework of outperforming opponents, MT has evolved into a more self-referenced and goal-based framework, evaluating performance using personal goals and standards regardless of any previous failures (Gucciardi, 2017). MT is important in itself as it is not a permanent mental state like grit, resolve, or determination, primarily due to the limitations of the latter constructs. MT is conceptually distinct from, but related to, psychological hardiness and psychological resistance. Psychological resilience refers to the capacity to recover and adapt positively following exposure to adversity, trauma, or significant stress (Fletcher and Sarkar, 2016). Unlike mental toughness, which involves proactive engagement during stress, psychological resilience is primarily a reactive process of bouncing back after disruption. A recent evidence by Denovan et al. (2023) confirms that while MT and psychological resilience are positively correlated, they load on distinct latent factors and predict unique variance in performance outcomes, supporting their treatment as separate constructs. Specifically, psychological resilience takes place after a setback, while MT occurs as an involvement in fixed moments with the awareness that a future effect or outcome is possible. For MT there is an element of active participation of support in possible adaptive and continual psychological engagement following a diverse range of stresses occurring across seldom endurance and gradual intensities and durations (Gucciardi, 2017). MT is different from grit as it accommodates multiple and even conflicting goals whereas grit has a singular focus of unwavering long-term commitment to one goal (Gucciardi, 2017). As such, MT serves as an enabling factor for effective emotional regulation and psychological resilience, both of which are critical mechanisms in the development and expression of athletic self-efficacy.

Self-efficacy, as described by Bandura (1991), exists as an important theoretical framework in regard to athletic involvement and sport success. Based on social cognitive theory, self-efficacy is defined as one's ability to carry out an action and reach the desired results when faced with problems or adversity (Rogowska et al., 2022). Also related to sport, self-efficacy is needed to focus athletes on their motivation and stamina with regard to problems (Kim et al., 2023). According to Rogowska et al. (2022), higher self-efficacy is understood to be related to more successful sport performance, as individuals who possess high self-efficacy are more likely to prevail in times of adversity, consider obstacles to be challenges to overcome, and can recover from tragedy. In addition, their study mentions that self-efficacy can explain how an athlete will regulate their behaviors, cope with stress, and apply coping strategies. All of which can influence the sport's overall success. Research shows self-efficacy directly predicts sport success (Hepler, 2016). This finding matches previous research (Hess, 2024; Nazarudin et al., 2025) suggesting that self-efficacy increases physical activity, persistence on a task, and effectiveness in applying mental imaging and goal-setting skills of an athlete. Therefore, with all this evidence available, self-efficacy is seen as a good effective psychological and behavioristic phenomenon, which establishes the relevant links between cognitive beliefs and behavioral activities in the area of sports.

Psychological resilience refers to the individual's ability to respond effectively and positively to challenges, trauma or considerable stress (Fletcher and Sarkar, 2013). It is more than simply the absence of psychological turmoil but an aactive process of preserving or regaining efficacy and well-being in the face of difficulty, challenge or hardship (Butler et al., 2006). Individuals who are resilient at the psychological level show cognitive flexibility, emotional control, and a tendency to view adversity as temporary and surmountable, helping them to maintain self-efficacy and performance under pressure (Hart, 2024). In sport contexts, psychological resilience is particularly vital, as it enables athletes are able to respond with motivation to perform well, handle competitive stress, recover from setbacks, and thus facilitate long range development of the athlete and in particular their psychological sustainability (Sarkar and Fletcher, 2014). Emotional regulation refers to the processes individuals use to influence which emotions they have, when they have them, and how they experience or express them (Gross, 2014). Emotional regulation includes strategies such as cognitive reappraisal, which is redefining a situation so as to alter its emotional meaning or effect, or expressive suppression, which involves hiding or holding in the display of emotions (Gross and John, 2003). Research indicates that successful emotional regulation, particularly cognitive reappraisal, is related to more favorable psychological outcomes, such as higher self-efficacy, reduced distress and improved wellbeing (Sha et al., 2022). In sport contexts, athletes who employ adaptive regulation strategies are better able to maintain focus, manage competitive stress, and sustain performance under pressure, positioning emotional regulation as a key mechanism through which dispositional traits like mental toughness influence performance-related beliefs.

Autonomy-supportive coaching is a relational style rooted in Self-Determination Theory (Deci and Ryan, 2012) wherein coaches foster athletes' sense of volition, choice, and personal agency by providing meaningful rationales, acknowledging feelings, offering options, and encouraging self-initiated behavior (Reynolds and McDonough, 2015). Their study asserts that rather than relying on controlling tactics or pressure, autonomy-supportive coaches create a psychologically safe environment that nurtures the basic needs for autonomy, competence, and relatedness–thereby enhancing intrinsic motivation, psychological well-being, and adaptive performance outcomes. Empirical studies confirm that this coaching approach is especially effective when combined with emotional involvement, as it strengthens the link between supportive behaviors and athletes' need satisfaction and self-determined motivation (Reynolds and McDonough, 2015).

## 3 Hypotheses

### 3.1 Mental toughness and psychological resilience

Mental toughness has become widely recognized as an important psychological attribute, particularly in sport, with which individuals (especially athletes) cope with pressure, challenges, and adversity in performance (Aditya et al., 2024). Along with aspects such as psychological resilience, mental toughness consists of confidence, consistency, control, and remaining motivated and focused on goals in the face of stress and adversity (Dorling and Bahr, 2024). These examples clearly show different features and traits that help foster psychological resilience. Resilience is defined as the capacity to bounce back after experiencing stress, adversity, and failure, and functions whereby an individual develops after facing adversity (Fletcher and Sarkar, 2016). There is empirical evidence that mentally tough athletes make positive appraisals of the stressful context, have affective stability, and consistently utilize adaptive coping strategies (Nüeztel, 2023). These adaptive patterns are also central features of psychological resilience. Mentally tough athletes are more likely to recover from injury, gradual performance decline, or competitive pressures, demonstrating resilience by maintaining a goal-oriented approach and emotional stability (Willis, 2018). In addition, research has begun to suggest that mental toughness has the potential to be a precursor of psychological resilience by supporting the internal arsenal for their recovery and high performance levels (Cowden et al., 2016). Therefore, mental toughness acts as a buffer to the negative consequences of stress and enhances psychological resilience-based functioning. Given this theoretical and empirical support, it is proposed that:

H1: Mental toughness has a significant positive effect on psychological resilience.

### 3.2 Mental toughness and emotional regulations

Mental toughness is not only a predictor of performance and perseverance but also closely tied to an individual's ability to manage internal states such as stress, anxiety, and emotional arousal (Soundara Pandian et al., 2023). One of the most important elements of mental toughness is emotional control–i.e., the ability to remain calm, focused, and confident, especially in an emotion - or pressure-filled situation (Crust, 2009). This connects mental toughness with emotional regulation, which refers to the processes of influencing which emotions one has, when one has them, and how one experiences and expresses them (Han et al., 2024). Athletes who are mentally tough have an easier time using adaptive emotional regulatory strategies, such as cognitive reappraisal, attentional control, and acceptance, allowing them to remain calm and perform optimally under pressure (Jannah et al., 2024). Research shows that mentally tough athletes report lower levels of emotional reactivity and are more likely to use constructive techniques for emotion regulation instead of maladaptive strategies like suppression or avoidance (Xin et al., 2024). Mental toughness helps athletes maintain emotional stability by regulating themselves when it comes to negative affect and being able to rebound quickly from emotionally distressing occurrences such as sub-par performance, criticism, or surprises (Adeyemi, 2025). This allows them to remain task-focused and behaviorally consistent despite fluctuations in their emotional state. Therefore, drawing on both theoretical perspectives and empirical findings, it is expected that:

H2: Mental toughness has a significant positive effect on emotional regulation.

### 3.3 Psychological resilience and performance self-efficacy

Psychological resilience refers to the capacity to adapt or recover following adversity, stress, or failure. It is a key component in establishing athletes' beliefs about their ability to succeed (Galli and Gonzalez, 2015). Psychological resilience provides the ability to concentrate, maintain emotional control, and direct effort toward achieving goals when the conditions may be more adverse and unpredictable, which often occurs in competitive situations (Palamarchuk and Vaillancourt, 2021). Performance self-efficacy is the belief that one can organize and execute the actions to produce a specific performance outcome (Bandura, 1991). This belief is multidimensional and is centered on mastery experiences, emotional state, and coping mechanisms. High levels of psychological resilience further contribute to this belief. Resilient athletes are more accustomed to seeing failure as transitory and ultimately surmountable, as opposed to an indicator of personal weakness. This cognitive appraisal allows for greater confidence in adapting to similar situations, thereby enhancing self-efficacy (Sarkar and Fletcher, 2014). Resilience is associated with performance self-efficacy in both studies and research and has been shown to positively predict self-efficacy in sports, education, and military training contexts (Bingöl et al., 2019). In addition, resilient athletes tend to demonstrate improved emotional regulation and problem-solving abilities, which may reinforce their belief in their ability to meet the performance demands (Kay and Merlo, 2020). As such, psychological resilience decreases vulnerability to the negative effects of stress and facilitates the internal resources that athletes need to pursue goals with general confidence. Given this evidence, the following hypothesis is proposed:

H3: Psychological resilience has a significant positive effect on performance self-efficacy.

### 3.4 Emotional regulation and performance self-efficacy

Emotional regulation—the degree to which emotional reactions can be monitored, judged, and changed—can also assist athletes in shaping their perceptions of, and reactions to, performance-related stressors (Gross, 2014). Athletes regularly encounter pressure, backsliding on learnings, disappointment from failures, and ever-shifting emotional states as they try to manage the demands of the sport. As a result, athletes' emotional regulation—most notably regulating feelings and thoughts of anxiety, frustration, and disappointment—contributes to being calm and focused in competitive situations to maintain their confidence and sense of clarity in managing a difficult performance context. Emotional stability facilitates clear thinking and perceived control–that is, the two core antecedents of self-efficacy (Al-Thunayan, 2025). The literature repeatedly has suggested that the degree of an athlete's emotional regulation skills (how the athlete regulates emotions) impacts their perceptions of stressful situations as manageable and how committed they are to performance goals (Nicholls et al., 2016). The use of adaptive strategies such as cognitive reappraisal or attentional control will buffer the impact of performance anxiety, leading to increased perceived capability to perform successfully. Additionally, emotional regulation aids in generating positive affect, which is positive to an athlete's motivation, task engagement, and increased belief they will perform successfully (Donoso et al., 2015). Through providing athletes a vehicle to utilize their emotions constructively, emotional regulation promotes and sustains self-efficacy and high-performance levels. Based on this theoretical and empirical support, the following hypothesis is proposed:

H4: Emotional regulation has a significant positive effect on performance self-efficacy.

### 3.5 Mediating role of psychological resilience

Mental toughness is a psychological quality that underlies an athlete's ability to keep attention, confidence, and composure during stress (Gucciardi, 2017). While mental toughness enhances performance-based outcomes, one of the main pathways confidence could develop performance self-efficacy is through psychological resilience (Eryilmaz et al., 2023). Psychological resilience is defined as the dynamic process of recovery and positive adaptation to adverse events, stressors, and despair (Galli and Gonzalez, 2015). Athletes who are mentally tough are likely to view stressors as challenges and opportunities rather than threats, thereby developing psychological resilience through repeated exposure to both strenuous situations and commonplace stressors associated with competition (Gucciardi, 2017). Over time, resilience builds psychological strength by allowing athletes to view challenges with increased comfort involuntarily while maintaining control of their emotions under pressure (Siebert, 2009). Psychological resilience supports performance self-efficacy, which is an athlete's belief in their ability to perform successfully, in a way that does not engender stress by sustaining a positive interpretation of possible stressful events, regulating their emotions, and employing adaptive coping strategies (Bingöl et al., 2019). Resilient athletes typically can continue even when they have failed while maintaining their belief in their performance ability (Galli and Gonzalez, 2015). Consequently, it is reasonable to hypothesize that psychological resilience acts as a mechanism through which mental toughness increases performance-related self-efficacy. Mental toughness generates a mindset that produces inner strength, whereas psychological resilience allows that strength to create adaptive responses and confidence levels in performance environments. Based on this reasoning, we propose the following hypothesis:

H5: Psychological resilience mediates the relationship between mental toughness and performance self-efficacy.

### 3.6 Mediating role of emotional regulations

Mental toughness provides athletes with the cognitive resources necessary to keep calm, remain confident, and maintain focus in times of adversity (Gucciardi, 2017). One of the key ways in which mental toughness can affect performance-related beliefs is via emotional regulation: the process by which we manage and modify emotional responses to stressors (Mutz et al., 2017). Athletes who are high in mental toughness are more likely to exert emotional control, one of the important facets of mental toughness (Aslam et al., 2021). Importantly, these individuals effectively control negative emotions (e.g., fear, anxiety, or frustration) that typically can compromise an individual's confidence and focus (Kashdan et al., 2011). For instance, these athletes may utilize adaptive emotion regulation strategies, such as cognitive reappraisal and attentional control, which allow them to maintain composure and manage their mental state during competitive pressure. In addition, effective emotional regulation can support performance self-efficacy–the belief about one's ability to perform well when pressured (Usán Supervía and Quílez Robres, 2021). Furthermore, athletes with emotional regulation abilities are able to maintain confidence in their ability to succeed, interpret stress as manageable or controllable, and persist in motivation to perform (Soflu et al., 2011). These findings demonstrate that emotional regulation can act as a psychological pathway between the internal strength associated with mental toughness and the individual's belief about his/her performance capabilities. Accordingly, we propose the following hypothesis:

H6: Emotional regulation mediates the relationship between mental toughness and performance self-efficacy.

### 3.7 Moderating role of autonomy-supportive coaching

Autonomy-supportive coaching is a coaching style that emphasizes choice, volition, and psychological empowerment by taking the perspective of the athlete, encouraging self-initiation, and limiting controlling actions (Burrell, 2016). This form of coaching is rooted in Self Determination Theory (SDT) and has been shown to promote athletes' internal motivation, well-being, and adaptive psychological functioning–especially in high/power situations (Amorose and Anderson-Butcher, 2015). Mental toughness is viewed as a stable psychological trait, but the athletic environment and the support of others, particularly coaches, can facilitate or hinder the expression of mental toughness in relation to important psychological outcomes such as psychological resilience and emotion regulation, respectively. An autonomy-supportive coach gives athletes a sense of autonomy and psychological safety and may advance the positive influence of mental toughness on psychological resilience (Mahoney et al., 2016). In that, mentally tough athletes may be better able to sustain psychological resilience when they have agency in making decisions, self-initiate decisions, and learn from these experiences in a non-pressurized environment (?). The coach's support helps to develop the athlete's own internal coping resource and promotes their adaptive appraisal and response to adversity. Similarly, it is anticipated that autonomy-supportive coaching will assist the development of mentally tough athletes' emotion regulation ability, based on the support of emotional awareness and reduction of negative influences from the external environment or pressures. In this way, an autonomy-supportive coach can develop the context for mentally tough athletes to regulate their emotions (Weinberg et al., 2011). When athletes feel understood and encouraged by their relationships with others, including coaches, an expectation exists that they will use emotion regulation skills, such as reappraisal and attention redirection (Davis and Davis, 2016), to optimize the emotional control that they have learned through mental toughness. Therefore, it is proposed that autonomy-supportive coaching strengthens the effects of mental toughness on both psychological resilience and emotional regulation.

H7a: Autonomy-supportive coaching positively moderates the relationship between mental toughness and psychological resilience.
H7b: Autonomy-supportive coaching positively moderates the relationship between mental toughness and emotional regulation.

**Figure 1:**
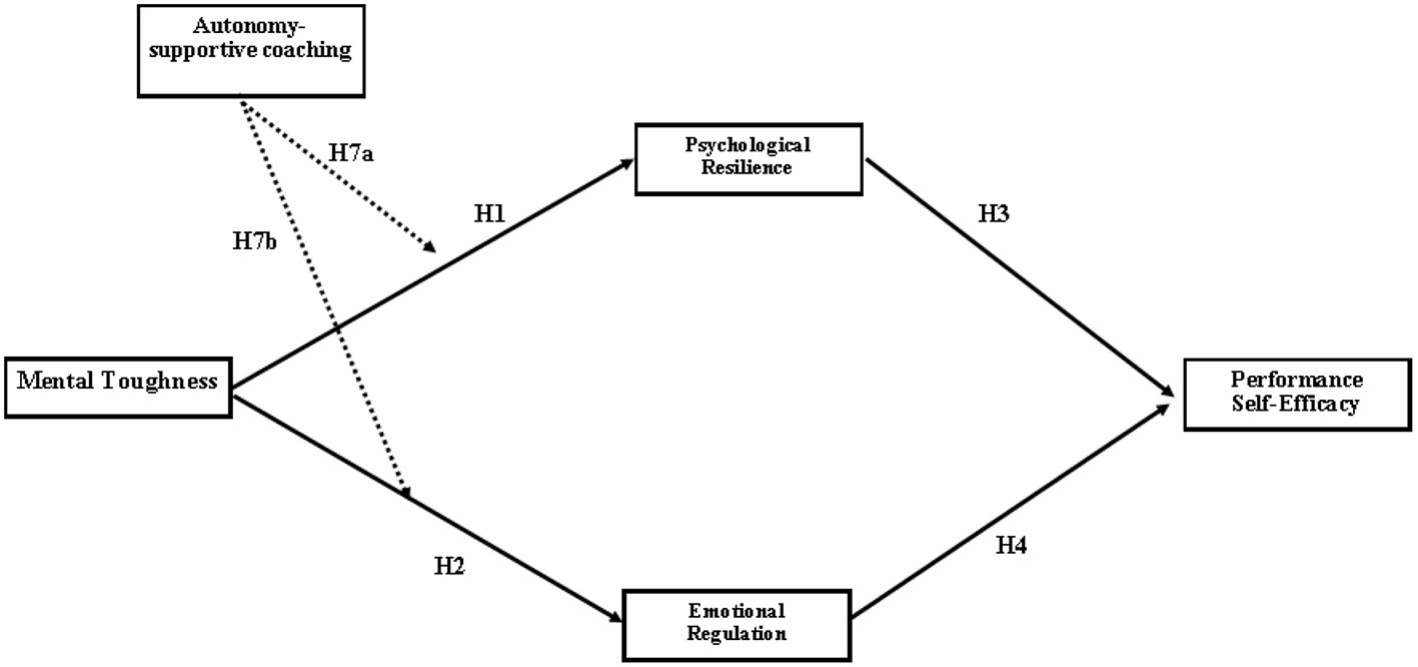
The proposed model.

Figure 1 shows the proposed model.

## 4 Methods

### 4.1 Data collection

This study used a quantitative research design to investigate proposed relationships among mental toughness, psychological resilience, emotional regulation, autonomy-supportive coaching, and self-efficacy. Data were collected through an online survey sent to athletes studying at various universities in China. The survey was open for a total of 45 days. A total of 1,000 questionnaires were sent to athletes via institutional email lists and athlete groups, with a view to collecting as many responses as possible during that time. This resulted in a total of 620 responses, with a response rate of 62%. However, after screening for usable responses, 581 usable responses were retained for analysis after removing any unusable responses. Participants were recruited from sports clubs and university sports departments in several different cities across China. For recruitment into the study, participants had to be a university student and an active participant in organized sporting activity for longer than one year. The sample included athletes from individual (e.g., track and field, swimming, martial arts; 42%) and team sports (e.g., basketball, volleyball, soccer; 58%). Competitive levels ranged from provincial (31%), national (47%), to international (22%). On average, athletes reported 8.3 years (SD = 3.1) of competitive experience. G^3.1^ was used to assess statistical power to ensure enough overall power to detect the hypothesized relationships in the study. The minimum sample size of this study was calculated as 246 based on a medium effect size, α = 0.05, power (1-β) = 0.95. Thus, the final sample size of 581 was adequate for testing the hypothesized relationships between the constructs using structural equation modeling, as well as the appropriation of this power to test moderation. Of the respondents, 58% identified as male, and 42% identified as female. In terms of age cohorts, 67% were 18–22 years old, 28% were 23–26 years old, and 5% were above 26 years of age. In terms of education level, 74% were athletes at the bachelor's level, 21% were at the master's level, and 5% were at the doctoral and other advanced level. This diverse sample provides a reliable foundation for examining the psychological constructs associated with athletic performance in the Chinese university context.

### 4.2 Measures

All scales were selected based on prior validation in athletic, strong psychometric properties, and conceptual alignment with our theoretical model. Where available, we prioritized scales previously used in similar sport psychology studies. The questionnaire consisted of two sections. The first section gathered demographic information such as age, gender, and years of athletic experience. The second section included validated scales to measure the main constructs. For each construct, a composite score was calculated as the mean of all items in the respective scale. All scales demonstrated strong internal consistency, with Cronbach's alpha and composite reliability values exceeding the recommended threshold of 0.70.

#### 4.2.1 Mental toughness

The athlete's Mental Toughness Questionnaire was adopted from Ergin et al. (2023) to assess mental toughness within sports settings. The questionnaire contains 14 items, which were assessed with 4-point Likert scale. Example items include: ‘I have what it takes to perform well while under pressure' and ‘Under pressure, I am able to make decisions with confidence and commitment' (1 = Totally False, 4 = Totally True).

#### 4.2.2 Psychological resilience

The athletes' psychological resilience measuring items were considered from Güngör et al. (2021). The questionnaire contains 6 items, which were assessed with 5-point Likert scale. Example items: “I tend to bounce back quickly after hard times” and “It does not take me long to recover from a stressful event” (1–5 Likert).

#### 4.2.3 Emotional regulation

Emotional regulation was measured with the defined items by Sha et al. (2022), containing 10 items which were assessed with 7-point Likert scale. Example items: “I control my emotions by changing the way I think about the situation I'm in” and “I control my emotions by not expressing them” (1–7 Likert).

#### 4.2.4 Self-efficacy

Self-efficacy was measured with the defined items, with 15 items (Chen et al., 2020) 15 items which were assessed with 5-point Likert scale. Example items: “I am confident I can perform well under pressure” and “I believe I can overcome obstacles to succeed” (1–5 Likert).

#### 4.2.5 Autonomy-supportive coaching

The Autonomy-supportive coaching measuring items were considered from Reynolds and McDonough (2015). The questionnaire contains 9 items, which were assessed with 7-point Likert scale. Example items: “I feel that my soccer coach provides me with choices and options” and “My coach encourages me to make my own decisions” (1-7 Likert).

### 4.3 Data analysis

To test the proposed relationships in the research model, Partial Least Squares Structural Equation Modeling (PLS-SEM) was utilized using SmartPLS 4.0. PLS-SEM was selected for this analysis for its ability to facilitate predictive, theory-building research; its ability to appropriately model complex models with multiple mediators and a moderator when the data do not conform to the assumptions of multivariate normality (Hair et al., 2021); and its ability to explain directly as well as indirectly and provide an outcome of predictions in research (to enhance research application). The analysis followed a two-step approach: the first step measured the measurement model to assess the reliability and validity of the constructs in the model through their indicators (factor loadings, Cronbach's alpha, composite reliability, average variance extracted [AVE]). After the measurement model was verified as meeting threshold levels for reliability and validity, the second step was to assess the structural model testing hypothesized paths, which were assessed with path coefficients, *t*-values, and *p*-values created from a bootstrapping procedure with 5000 re-samples. Finally, R^2^ values, effect sizes (f^2^), predictive relevance (Q^2^), and the overall explanatory power/predictive power of the model were identified. The moderating effects of autonomy-supportive coaching were also tested through interaction terms within the PLS framework.

## 5 Results

### 5.1 Data screening

Before conducting the measurement model and testing the hypotheses, a data screening process was performed to affirm the quality of the data and appropriateness of the dataset. Harman's single factor test was used to measure Common Method Bias (CMB), and it found that the first factor explained 41.574% of total variance, which is below the 50% fragmentation threshold, indicating no significant CMB concerns (Podsakoff et al., 2003). Descriptive statistics reported mean values for the items that reflected a range from 2.040 to 2.549 and standard deviations from 0.698 to 9.96, confirming enough variability in the sample. Skewness and kurtosis values for all items were within the acceptable level of ±2, indicating no significant deviations from normality. Multicollinearity, as measured with the Variance Inflation Factor (VIF) measurement, had values ranging from 1.353 to 3.447, well below the commonly accepted rule of thumb of 5, confirming the absence of multicollinearity without any significance. Full descriptive statistics are presented in Table 1.

**Table 1:** Descriptive statistics and distribution characteristics of study constructs.

**Construct**	**Mean**	**Std. deviation**	**Skewness**	**Kurtosis**
Mental toughness	2.040	0.698	0.471	-0.524
Psychological resilience	2.528	0.755	0.288	-0.015
Emotional regulation	3.534	0.996	0.247	0.081
Performance self-efficacy	2.549	0.739	0.183	-0.386
Autonomy-supportive coaching	3.541	1.318	0.166	-0.602

### 5.2 Measurement model

**Figure 2:**
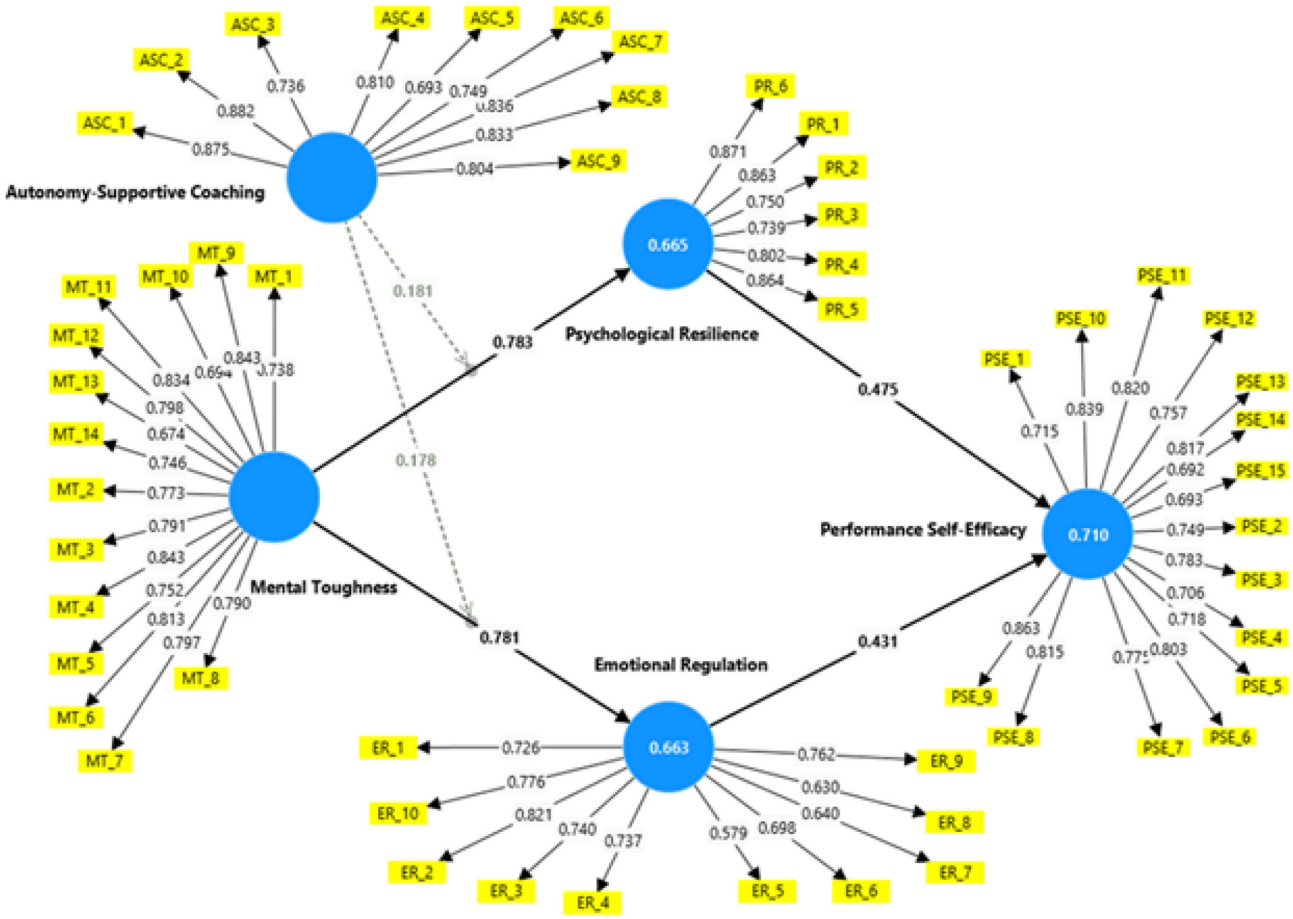
Measurement model with factor loadings and construct reliabilities.

Reliability and convergent validity are two important indicators of measurement quality in structural equation modeling. Reliability estimates the internal consistency aspects of a construct and can be calculated by Cronbach's alpha, composite reliability (CR), and rho_A; generally, a threshold of 0.70 or higher is deemed an acceptable reliability level (van Rens et al., 2021). By comparison, in this model, we generally used all three reliability indices, typically used Cronbach's alpha, CR, and rho_A to determine the reliability of the three constructs. Convergent validity indicates the degree to which items of a construct correlate and represent the same underlying concept and is typically evaluated through the average variance extracted (AVE) which shows the amount of variance a construct captures from its indicators. AVE scores above 0.50 demonstrate that a construct captures more than 50% of the variance from its indicators. Based on the results of the reliability indices, shown in Table 2, the reliability indices for the constructs exceeded the cut-off levels all three indices (Cronbach's alpha, CR, and rho_A) were greater than 0.70 and all AVE values were above 0.50, indicating the model has reliability and validity for all three constructs. In summary, all AVE scores support the reliability and validity for all three constructs, all factor loadings were above 0.70 (see Figure 2), further supporting convergent validity for all three constructs. These results collectively provide a robust foundation for subsequent structural analysis (Table 2).

**Table 2:** Reliability and convergent validity indices (Cronbach's α, CR, AVE).

**Construct**	**α**	**CR (rho_a)**	**CR (rho_c)**	**AVE**
Autonomy-supportive coaching	0.935	0.940	0.942	0.647
Emotional regulation	0.892	0.900	0.912	0.510
Mental toughness	0.950	0.952	0.956	0.607
Performance self-efficacy	0.951	0.954	0.956	0.595
Psychological resilience	0.899	0.907	0.923	0.667

Discriminant validity assesses how unique a construct is from the other constructs in the model, supporting the idea that the construct represents phenomena that are not represented by other variables. Discriminant validity is evaluated by assessing whether the square root of the average variance extracted (AVE) for each construct exceeds the construct's correlations with other constructs or through other ways, such as the Fornell and Larcker (1981) criterion. The Heterotrait-Monotrait ratio (HTMT) may also be used. In accordance with the results presented in Table 3, indicate the threshold necessary is satisfied. The square roots of the AVE from each construct are greater than the inter-construct correlations, and the HTMT values, which should be below the cut-off of 0.90, were below that observed (Henseler et al., 2015). These findings confirm adequate discriminant validity, indicating that the constructs are conceptually distinct and the measurement model is well specified.

**Table 3:** Discriminant VALIDITY: SQUARE ROOTS of AVE (diagonal) vs. Inter-construct correlations (below diagonal) and HTMT ratios (above diagonal).

**Construct**	**1**	**2**	**3**	**4**	**5**
1. Autonomy-supportive coaching	0.804				
2. Emotional REGULATION	0.068	0.794			
3. Mental toughness	0.053	0.713	0.779		
4. Performance self-efficacy	0.017	0.777	0.800	0.772	
5. Psychological resilience	0.051	0.728	0.694	0.749	0.817
**HTMT**	**1**	**2**	**3**	**4**	**5**
1. Autonomy-supportive coaching					
2. Emotional regulation	0.070				
3. Mental toughness	0.054	0.854			
4. Performance self-efficacy	0.043	0.840	0.839		
5. Psychological resilience	0.049	0.806	0.854	0.848	

### 5.3 Confirmatory factor analysis

The measurement model was evaluated using confirmatory factor analysis (CFA) to assess overall model fit prior to structural path estimation. As shown in Table 4, multiple goodness-of-fit indices were examined. The Comparative Fit Index (CFI) = 0.943 and Tucker-Lewis Index (TLI) = 0.936 exceed the recommended threshold of 0.90 for good fit (Hu and Bentler, 1998). Hu and Bentler (1998) recommend a cutoff value of .08 for SRMR; and 0.06 for RMSEA however; the value RMSEA should not exceed 0.08. The Root Mean Square Error of Approximation (RMSEA) = 0.074 falls below the 0.08 cutoff, indicating close fit. The X^*^/df ratio of 1.069 is well below the upper limit of 5.0, further supporting model adequacy (Lomax, 2004). Collectively, these fit indices indicate that the measurement model fits the data well, supporting the validity of the latent structure and justifying progression to structural model testing.

**Table 4:** Goodness-of-fit values.

**Fit indices**	**Recommended value**	**Source**	**Estimated model**
Chi-square		(Hu and Bentler, 1998; Lomax, 2004)	1460.823
*P* value	In-significant		0
ChiSqr/df	≤ 5		1.069
RMSEA	≤ 0.06		0.011
GFI	≥0.90		0.918
SRMR	≤ 0.08		0.024
NFI	≥0.90		0.935
TLI	≥0.90		0.927
CFI	≥0.90		0.928

### 5.4 Structural model

Once the reliability and validity of the measurement model had been confirmed, the structural model was evaluated to examine the hypothesized relationships between the constructs. The path coefficients, *t*-values, and *p*-values obtained through bootstrapping with 5,000 resamples confirmed the significance of all proposed hypotheses.

#### 5.4.1 The relationship between mental toughness, psychological resilience and emotional regulation

Mental toughness had a significant positive effect on psychological resilience (β = 0.783, *t* = 51.136, *p* < 0.001) and emotional regulation (β = 0.781, *t* = 48.71, *p* < 0.001). Thus, H1 and H2 are accepted.

#### 5.4.2 The relationship between psychological resilience, emotional regulation, and performance self-efficacy

Psychological resilience (β = 0.475, *t* = 15.982, *p* < 0.001) and emotional regulation (β = 0.431, *t* = 14.202, *p* < 0.001) significantly impacted performance self-efficacy. Thus, H3 and H4 are supported.

#### 5.4.3 Mediation effects of psychological resilience and emotional regulation

Both psychological resilience (β = 0.372, *t* = 15.179, *p* < 0.001) and emotional regulation (β = 0.337, *t* = 12.845, *p* < 0.001) significantly mediated the relationship between mental toughness and performance self-efficacy. Thus, H5 and H6 are supported.

#### 5.4.4 Moderating effects of autonomy-supportive coaching

**Figure 3:**
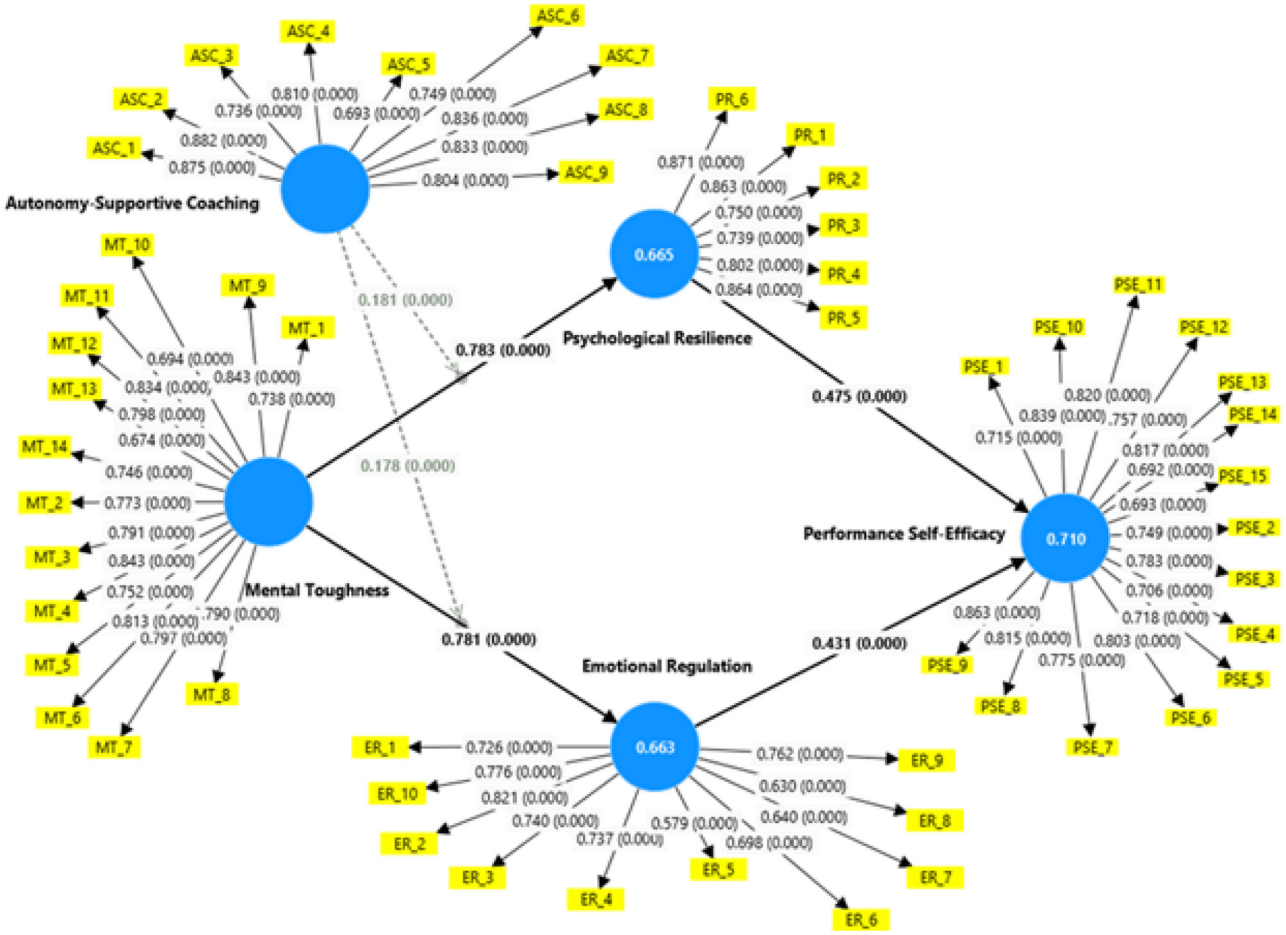
Final structural model with path coefficients and significance levels.

Additionally, autonomy-supportive coaching positively moderated the effect of mental toughness on psychological resilience (β = 0.181, *t* = 4.542, *p* < 0.001) and moderated its effect on emotional regulation (β = 0.178, *t* = 4.512, *p* < 0.001). Thus, H7a and H7b are accepted. The model explained substantial portions of variance: *R*^2^ = 0.663 for emotional regulation, *R*^2^ = 0.710 for performance self-efficacy, and *R*^2^ = 0.665 for psychological resilience, indicating moderate to substantial explanatory power. In addition, *Q*^2^ = 0.335 for emotional regulation, *Q*^2^ = 0.420 for performance self-efficacy, and *Q*^2^ = 0.440 for psychological resilience confirmed the predictive relevance of the model. These results, as presented in Table 5 and Figure 3, demonstrate strong empirical support for the proposed theoretical framework.

**Table 5:** Structural model results: direct, indirect, and moderated effects.

**Path**	**Beta**	***t*-value**	***p*-value**	**Supported**
H1. Mental toughness → Psychological resilience	0.783	51.136	0.000	Yes
H2. Mental toughness → Emotional regulation	0.781	48.710	0.000	Yes
H3. Psychological RESILIENCE → Performance self-efficacy	0.475	15.982	0.000	Yes
H4. Emotional regulation → Performance self-efficacy	0.431	14.202	0.000	Yes
H5. Mental toughness → Psychological resilience → Performance self-efficacy	0.372	15.179	0.000	Yes
H6. Mental toughness → Emotional regulation → Performance self-efficacy	0.337	12.845	0.000	Yes
H7a. Autonomy-supportive coaching × Mental toughness → Psychological resilience	0.181	4.542	0.000	Yes
H7b. Autonomy-supportive coaching × Mental toughness → Emotional regulation	0.178	4.512	0.000	Yes
		**Emotional regulation**	**Performance self-efficacy**	**Psychological resilience**
*R* ^2^		0.663	0.710	0.665
*Q* ^2^		0.335	0.420	0.440

**Figure 4:**
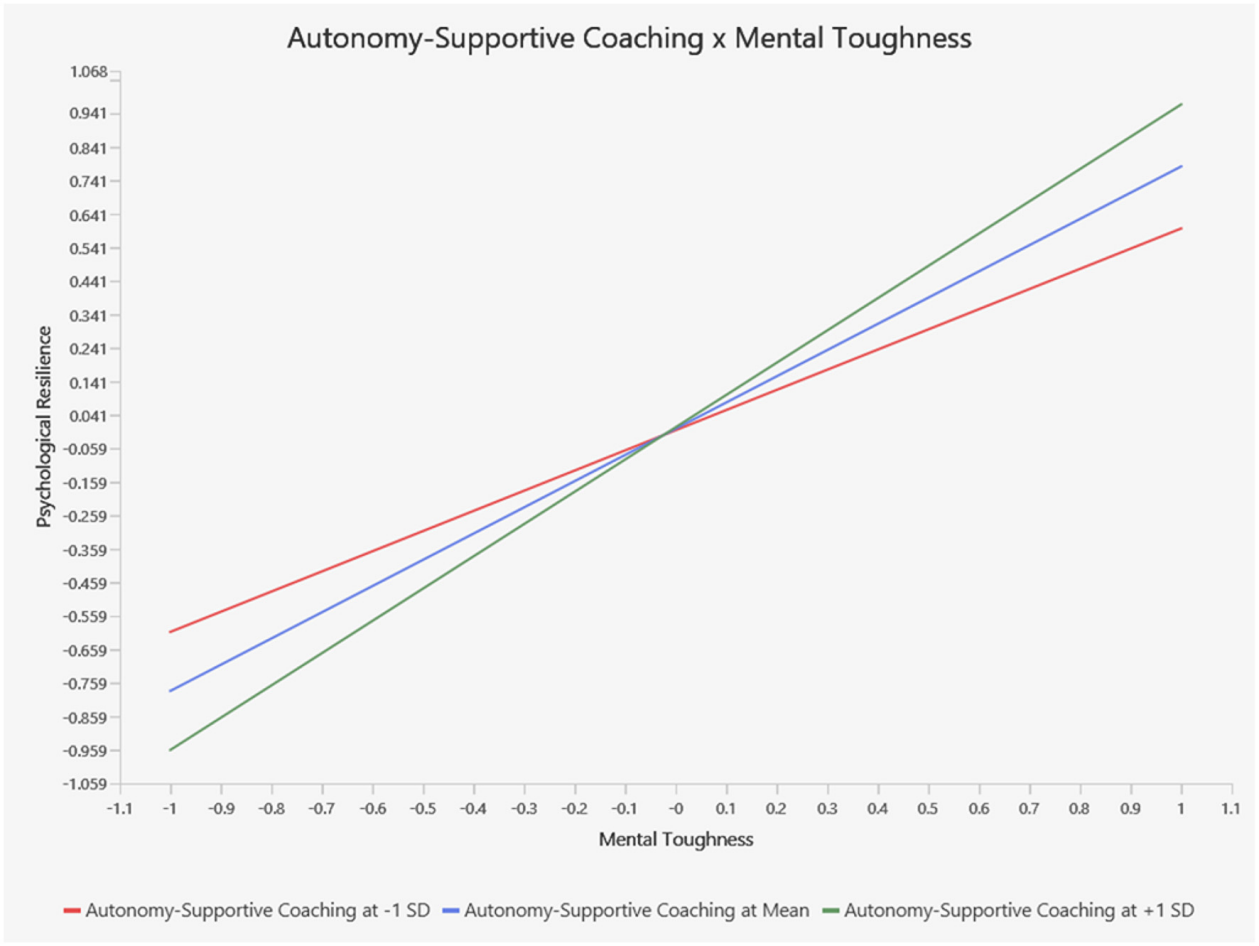
Moderating effect of autonomy-supportive coaching on the relationship between mental toughness and psychological resilience.

Figure 4 illustrates the moderating role of autonomy-supportive coaching on the relationship between mental toughness and psychological resilience. As shown, when autonomy-supportive coaching is low (see the red line), athletes exhibit a low level of psychological resilience. However, as autonomy-supportive coaching increases (see the green line), athletes' level of psychological resilience increases.

**Figure 5:**
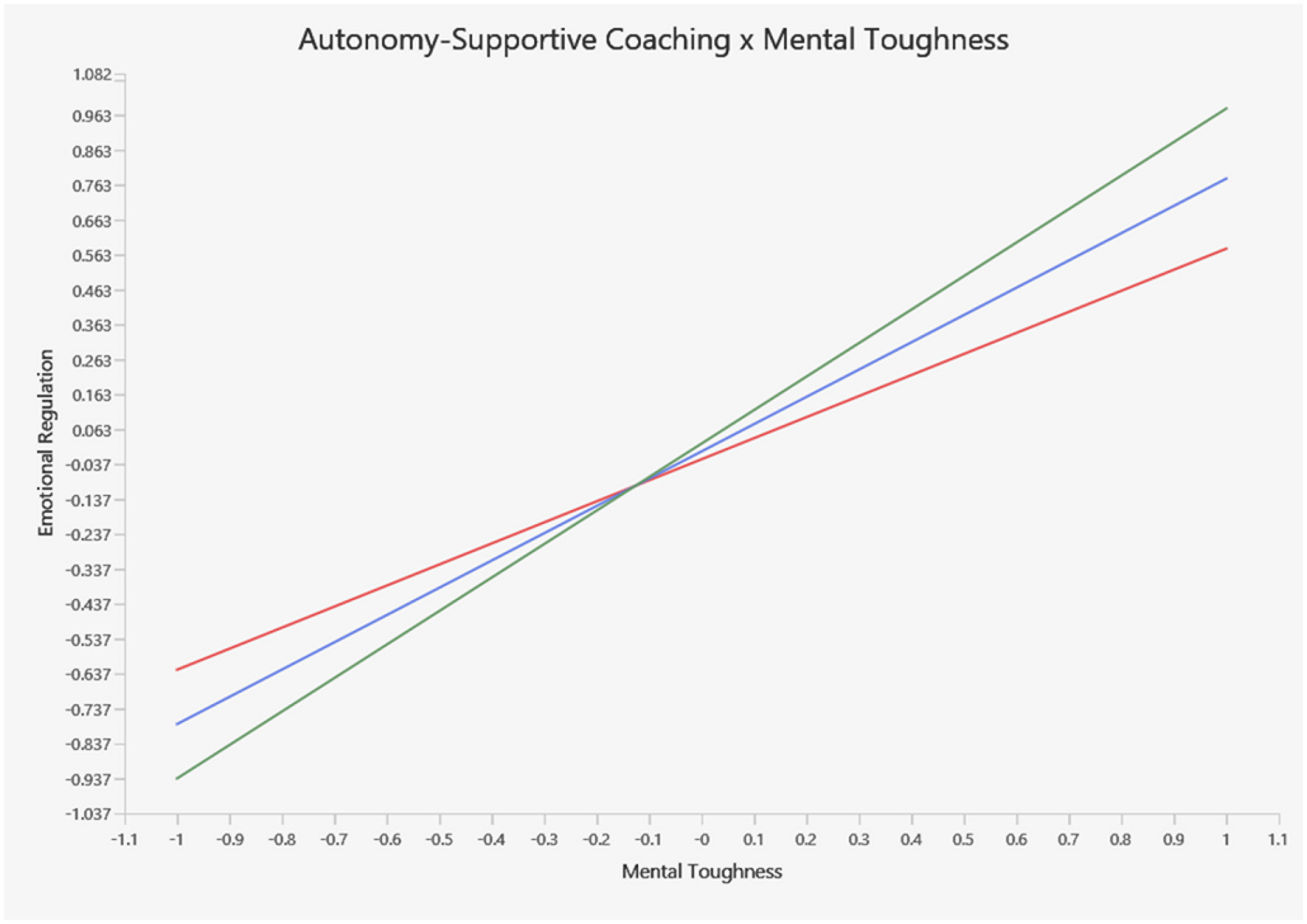
Moderating effect of autonomy-supportive coaching on the relationship between mental toughness and emotional regulation.

Figure 5 illustrates the moderating role of autonomy-supportive coaching on the relationship between mental toughness and emotional regulation. As shown, when autonomy-supportive coaching is low (see the red line), athletes exhibit a low level of emotional regulation. However, as autonomy-supportive coaching increases (see the green line), athletes' level of emotional regulation increases.

## 6 Discussion

Individuals in elite sport and education regularly deal with psychological pressures that threaten their emotional stability and ability to persist. Prior research has theorized about performance improvements associated with mental toughness (MT); there is limited theoretical and empirical exploration regarding how MT influences self-efficacy through important psychological mechanisms like emotional regulation (ER) and psychological resilience (PR). Most models of mental toughness have overlooked the variables of ER and PR under a single model and failed to include context for how variances such as autonomy-supportive coaching (ASC) may moderate the relationship. Given these gaps, this study presented the following questions: (1) Do mental toughness affect emotional regulation, psychological resilience, and performance self-efficacy? (2) Do emotional regulation and psychological resilience mediate the relationship between mental toughness and performance self-efficacy? (3) Does autonomy-supportive coaching moderate the influence of mental toughness on emotional regulation and psychological resilience? The structural model displayed substantial explanatory capability with R^2^=0.663, R^2^=0.710, and R^2^=0.665 for emotional regulation, psychological resilience, and performance self-efficacy, respectively. The model also displayed Q^2^=0.335 for ER, Q^2^=0.42 for PR, and Q^2^=0.44, indicating sufficient predictive relevance and prediction judgment for the model.

### 6.1 Mental toughness, psychological resilience, and emotional regulation

The study provided evidence that mental toughness (MT) was statistically and meaningfully positively associated with psychological resilience (PR) and emotional regulation (ER), suggesting that higher levels of mental toughness make it easier for an individual to regulate their emotions and recover from setbacks. This finding suggests that mental toughness provides the important psychological components that allow an individual to maintain control, respond to stress, and engage in behavior consistent with their goals in demanding circumstances, which are important qualities for both academic and competitive contexts. The positive and significant relationship between MT and PR coincides with the conceptualization that mental toughness promotes a sense of fortitude in a person's ability to maintain focus and optimism in challenging events. In the same vein, the positive and significant relationship with ER indicates that mentally tough individuals can manage their emotional responses to things such as anxiety, frustration, or fear, and continue to focus on their goals. These results support previous research (Mutz et al., 2017; Arora et al., 2022) that suggests that mental toughness supports resilience and emotional regulation, which represent two of the anchors of resilience. In conclusion, these findings provide further support for the theoretical perspective that mental toughness is an important contributor to positive psychological functioning and an important precursor to adaptive performance outcomes.

### 6.2 Psychological resilience, emotional regulation, and performance self-efficacy

The results further support both PR and ER having a positive effect on PSE, indicating the impact PR and ER have on individuals' confidence that they can successfully perform tasks. This leads us to think that if an individual can manage their emotions while recovering from adversity, they are more likely to believe in their capabilities. This is consistent with PR and ER because managing one's emotions means one spends less time stressing out or dwelling on setbacks, and hence can keep their focus on goal attainment. Resilient individuals are able to persist through challenges in order to stay motivated enough to maintain self-efficacy. PR and PSE have a positive relationship. This makes sense based on Bandura (1997) theory and its identification of overcoming adversity as one component of sources of efficacy beliefs. Past research also supports the influence ER has on PSE (Etherton et al., 2022). So, together, these findings reinforce the notion that psychological strengths play a role in developing a belief in one's abilities and capacity to achieve targeted outcomes.

### 6.3 Mediating effects of psychological resilience, emotional regulation

Apart from its direct effects, it was found that psychological resilience (PR) and emotional regulation (ER) act as strong mediators between mental toughness (MT) and performance self-efficacy (PSE). This suggests that MT's impact on PSE not only occurs directly through the enhancement of key constructs, but indirectly through its contribution to more resilient individuals (who recover more quickly from adversity) and emotionally regulated individuals (who better manage their emotions in a distressing manner), which aid in elevating self-efficacy and confidence in difficult tasks. These assessments of mediation show how mental toughness impacts self-efficacy by developing or adding to internal capacities to help manage adversity and emotional turmoil with success. These findings add a more complete understanding of mental toughness as an essential characteristic or trait developed and used by individuals that enhances their performance self-efficacy by preparing the development of psychological adaptability and emotional competence.

### 6.4 Moderating effects of autonomy-supportive coaching

The research also shows that autonomy-supportive coaching (ASC) positively and significantly moderates the relationship between mental toughness (MT) and emotional regulation (ER), and psychological resilience (PR). When individuals perceive their coaches or mentors to be autonomy-supportive by allowing self-initiation, providing meaningful rationales, and being willing to accept an individual's perception, the positive relationship between mental toughness and key psychological outcomes is amplified. In other words, supportive autonomy increases the extent to which mentally tough individuals can regulate their own emotions and return to a baseline state after challenges. This interaction suggests the importance of contextual support for psychological functioning. Supportive autonomy is also in accordance with Self-Determination Theory (Deci and Ryan, 2012), which posits that this kind of coaching fosters intrinsic motivation and psychological development, thereby enhancing the impact of personal traits like mental toughness. Previous studies (Zhang et al., 2025; Cece et al., 2022) also indicate that autonomy-supportive coaching contributes to athletes' emotional well-being and resilience. The moderation effects of autonomy-supportive coaching, while statistically significant, represent a small-to-medium practical effect, suggesting that coaching style meaningfully amplifies, but does not override, the role of mental toughness. In real-world terms, athletes high in mental toughness gain substantially more resilience and emotional regulation when coached in autonomy-supportive environments, highlighting the value of coach training in supportive communication. Thus, these results not only affirm the importance of MT as a psychological resource but emphasize the importance of fostering social contexts that encourage the recognition and amplification of one's internal strengths.

## 7 Implications and limitations

### 7.1 Theoretical implications

This study has significant theoretical implications. The substantial direct and indirect influence of mental toughness (MT) on performance-related self-efficacy (PSE), through psychological resilience (PR) and emotional regulation (ER), increases our understanding of the psychological mechanisms related to performance outcomes. By incorporating elements of MT, PR, ER, and PSE into the same model, the current investigation moves the theoretical base of MT research forward, considering MT as a foundational trait that may impact performance outcomes through psychological mechanisms, and not just through direct paths. Furthermore, the support for ER and PR as mediators further suggests the conceptual importance of PR and ER in educational psychology, and, given prior theories, including Bandura's Social Cognitive Theory, the current findings also support existing frameworks around psychological constructs of resilience and emotional regulation. The significant conceptual role of autonomy-supportive coaching (ASC) extends the boundaries of Self-Determination Theory (Cowden et al., 2016) and demonstrates how contextual support can impact individual traits toward positive psychological outcomes. Overall, the findings provide support in demonstrating the multidimensionality of psychological performance frameworks and provide future theoretical frameworks with an impetus to account for personal factors, as well as contextual factors, in explaining high and low-performance behavior. Our findings also enrich SDT by illustrating how autonomy-supportive contexts amplify dispositional traits, and align with Lazarus and Folkman's transactional model (Lazarus and Folkman, 1984) by showing how MT shapes stress appraisal (via ER/PR), thereby influencing coping efficacy.

### 7.2 Practical implications

The results of this study offer several practical implications, particularly for coaches, educators, and performance professionals. First, the significant role of mental toughness (MT) in enhancing emotional regulation (ER), psychological resilience (PR), and ultimately performance self-efficacy (PSE), suggests that training programs should focus not only on technical skills but also on developing mental strength. For example, in competitive sports, coaches can incorporate mental toughness exercises—such as goal-setting, visualization, and stress exposure training—to help athletes better manage pressure and recover from setbacks. Second, the findings highlight the importance of emotional regulation and resilience training as part of performance development. In academic settings, for instance, students facing exam stress or failure may benefit from interventions that teach coping strategies and emotional awareness to enhance confidence and performance. Third, the positive moderating role of autonomy-supportive coaching (ASC) underscores the need for leadership approaches that foster autonomy, respect, and encouragement. For example, a coach who provides athletes with choices in training routines, listens to their perspectives, and offers meaningful feedback is likely to strengthen the psychological impact of their athletes' mental toughness. For instance, coaches could integrate structured autonomy-supportive practices such as (1) offering choices in training drills, (2) using informational language (e.g., “This drill helps build endurance for late-game performance”) rather than controlling directives, and (3) holding regular one-on-one reflection sessions to validate athletes' perspectives—all evidence-based strategies from SDT-based interventions (Mahoney et al., 2016). Overall, these findings suggest that integrating both personal and social development strategies can lead to more effective and sustainable performance outcomes across various high-pressure domains. We recognize that implementing ASC may be challenging in high-pressure, results-driven environments where time constraints, institutional expectations, or coach training gaps limit autonomy-supportive practices. Systemic support such as coach education programs and organizational policies, may be necessary to enable sustainable adoption.

### 7.3 Limitations

Despite its theoretical and applied merits, the current research has several limitations. First, the cross-sectional design precludes causal inference regarding directionality among the relationships between mental toughness, psychological resilience, emotional regulation, and self-efficacy. Future research is encouraged to employ longitudinal panel designs (e.g., tracking athletes over a competitive season) or experimental interventions (e.g., randomized autonomy-supportive coach training) to test for causality and malleability of these pathways. Second, the sample consisted only of Chinese university athletes, a population experiencing dual academic-athletic pressures within a collectivist cultural context that may value obedience over autonomy (Song et al., 2024; Zhou and Zhang, 2024). These contextual factors could amplify the role of autonomy-supportive coaching or limit mental toughness expression, decreasing generalizability to professional elites, youth sport participants, or individualistic cultures. Future studies would benefit from replicating this model with alternative cultural, age, and competitive-level groups to establish boundary conditions and enhance external validity. Third, sole reliance on self-report measures–while common in sport psychology research–risks biases like social desirability (e.g., athletes over-report mental toughness or perceived coaching support) and shared method variance, despite our statistical tests (e.g., Harman's single-factor test, marker-variable technique). Triangulation with objective indicators such as coach ratings, behavioral observations, performance metrics, or physiological measures (e.g., heart rate variability, cortisol level, EEG for stress tasks), would increase construct validity in future studies. Finally, while our model emphasizes emotional regulation and psychological resilience as theoretically driven mediators, other mechanisms such as intrinsic motivation, stress appraisal, or coping flexibility, could also link mental toughness to self-efficacy. Also, possible subgroup differences based on gender, type of sport (individual vs. team), or level of competition are yet untested; future studies may investigate such moderators to sharpen the model's generalizability.

## 8 Conclusion

The present study sought to fill the existing gap in our understanding of the psychological processes related to how MT affects performance self-efficacy, through looking at the mediating effects of emotional regulation and psychological resilience. Notably, this study is among the first to empirically demonstrate that autonomy-supportive coaching functions as a critical contextual amplifier of mental toughness, transforming internal strength into adaptive psychological outcomes. Data were collected online via a sample of 581 university athletes in China, and SEM was used to analyze the interrelationships. Overall, the findings showed that MT had a positive and significant effect on ER and PR, which, in turn, had a positive effect on PSE. Furthermore, ER and PR were also shown to significantly mediate the relationship between MT and PSE, and ASC positively moderated the relationships between MT and ER and PR. These results had significant theoretical implications by advancing integrated models of psychological performance, indicating the need for more research on the role of contextual support to improve individual traits. Practically, these results indicated that coaches and practitioners in sports need to consider athletes' development of their mental toughness as well as building autonomy-supportive environments that will help them develop the emotional and resilient capacities necessary that enhance self-efficacy. Nonetheless, the study's cross-sectional design and culturally specific sample of Chinese university athletes limit the generalizability of the findings, suggesting that future research should explore longitudinal designs and diverse populations to validate and extend these insights.

## Data Availability

The raw data supporting the conclusions of this article will be made available by the authors, without undue reservation.

## References

[B1] Adeyemi A. E. (2025). Mental Toughness, Emotion Expressivity and Psychological Well-being (PhD thesis). Manchester Metropolitan University, Manchester, UK.

[B2] Aditya R. S. Rahmatika Q. T. Solikhah F. K. AlMutairi R. I. Alruwaili A. S. Astuti E. S. . (2024). “Mental toughness may have an impact on athlete's performance: systematic review,” in Retos: Nuevas Tendencias en Educación Fí*sica, Deporte y Recreación*, 328–337.

[B3] Aizava P. V. S. dos Santos Oliveira I. F. de Oliveira D. V. Garcia W. F. (2024). Relationships between self-efficacy and high-performance sport: a systematic review. Paidéia (Ribeir ao Preto) 34:e3412. doi: 10.1590/1982-4327e3412

[B4] Al-Thunayan A. M. (2025). The Controlled Mind: A Theory of Mental Clarity and Self-Leadership.

[B5] Amorose A. J. Anderson-Butcher D. (2015). Exploring the independent and interactive effects of autonomy-supportive and controlling coaching behaviors on adolescent athletes' motivation for sport. Sport, Exerc. Perform. Psychol. 4:206. doi: 10.1037/spy0000038

[B6] Anstiss P. A. Meijen C. Marcora S. M. (2020). The sources of self-efficacy in experienced and competitive endurance athletes. Int. J. Sport Exerc. Psychol. 18, 622–638. doi: 10.1080/1612197X.2018.1549584

[B7] Arora T. Grey I. Östlundh L. Alamoodi A. Omar O. M. Lam K.-B. H. . (2022). A systematic review and meta-analysis to assess the relationship between sleep duration/quality, mental toughness and resilience amongst healthy individuals. Sleep Med. Rev. 62:101593. doi: 10.1016/j.smrv.2022.101593 35462348

[B8] Aslam S. Saleem S. Mahmood Z. (2021). Mental toughness and mental health problems in doctors: a mediating role of emotion regulation. Khyber Med. Univers. J. 13, 10–14. doi: 10.35845/kmuj.2021.19923

[B9] Bandura A. (1991). Social cognitive theory of self-regulation. Organ. Behav. Hum. Decis. Process. 50, 248–287. doi: 10.1016/0749-5978(91)90022-L

[B10] Bandura A. (1997). Self-Efficacy: The Exercise of Control, Volume 11. Dallas, TX: Freeman.

[B11] Bandura A. (2023). “Cultivate self-efficacy for personal and organizational effectiveness,” in Principles of Organizational Behavior: The Handbook of Evidence-Based Management, 3rd Edn (Hoboken, NJ), 113–135.

[B12] Bingöl T. Y. Batik M. V. Hosoglu R. Firinci Kodaz A. (2019). Psychological resilience and positivity as predictors of self-efficacy. Asian J. Educ. Train. 5, 63–69. doi: 10.20448/journal.522.2019.51.63.69

[B13] Burrell C. J. (2016). The Relationship of Perceived Autonomy-Supportive Coaching Behavior with Motivation Among High School Athletes (Master's thesis). The University of North Carolina at Greensboro, Greensboro, NC, United States.

[B14] Butler L. D. Morland L. A. Leskin G. A. (2006). “Psychological resilience,” in Psychology Terror (Oxford; New York, NY), 400.

[B15] Cece V. Guillet-Descas E. Tessier D. Martinent G. (2022). Athletes' motivational and emotional outcomes related to a need-supportive intervention in intensive training centers. J. Appl. Sport Psychol. 34, 1206–1226. doi: 10.1080/10413200.2021.1941425

[B16] Çeetin M. K. Kumcu R. Çeelik M. (2025). Mental toughness in athletes: a comprehensive review. KARAMANOğLU MEHMETBEY ÜNİVERSİTESİ ULUSLARARASI BEDEN EğİTİMİ VE SPOR BİLİMLERİ DERGİSİ 2, 1–12.

[B17] Çetin M. K. Kumcu R. Çelik M. (2025). Mental toughness in athletes: a comprehensive review. KARAMANOGLU MEHMETBEY ÜNIVERSITESI ULUSLARARASI BEDEN EGITIMI VE SPOR BILIMLERI DERGISI 2, 1–12.

[B18] Chen X. Qiu N. Chen C. Wang D. Zhang G. Zhai L. (2020). Self-efficacy and depression in boxers: a mediation model. Front. Psychiatry 11:00791. doi: 10.3389/fpsyt.2020.00791 33132920 PMC7550717

[B19] Cowden R. G. Meyer-Weitz A. Oppong Asante K. (2016). Mental toughness in competitive tennis: relationships with resilience and stress. Front. Psychol. 7:320. doi: 10.3389/fpsyg.2016.00320 27014132 PMC4791384

[B20] Crust L. (2009). The relationship between mental toughness and affect intensity. Pers. Individ. Dif. 47, 959–963. doi: 10.1016/j.paid.2009.07.023

[B21] Davis P. A. Davis L. (2016). “Emotions and emotion regulation in coaching,” in The Psychology of Effective Coaching and Management, 285–306.

[B22] Deci E. L. Ryan R. M. (2012). “Self-determination theory,” in Handbook of Theories of Social Psychology (Thousand Oaks, CA), 416–436.

[B23] Denovan A. Dagnall N. Drinkwater K. (2023). Examining what mental toughness, ego resiliency, self-efficacy, and grit measure: an exploratory structural equation modelling bifactor approach. Curr. Psychol. 42, 22148–22163. doi: 10.1007/s12144-022-03314-5

[B24] Donoso L. M. Demerouti E. Hernández E. G. Moreno-Jimenez B. Cobo I. C. (2015). Positive benefits of caring on nurses' motivation and well-being: a diary study about the role of emotional regulation abilities at work. Int. J. Nurs. Stud. 52, 804–816. doi: 10.1016/j.ijnurstu.2015.01.002 25627792

[B25] Dorling J. Bahr M. (2024). Mental Toughness in Sports People. (Charlottesville, VA).

[B26] Duckworth A. L. Peterson C. Matthews M. D. Kelly D. R. (2007). Grit: perseverance and passion for long-term goals. J. Pers. Soc. Psychol. 92:1087. doi: 10.1037/0022-3514.92.6.1087 17547490

[B27] Ergin R. Çakir G. Isik U. (2023). Mediator role of perceived stress in the relationship between positive/negative emotions and mental toughness. Akdeniz Spor Bilimleri Dergisi 6, 37–51. doi: 10.38021/asbid.1161949

[B28] Eryilmaz A. Yildirim-Kurtulus H. Doenyas C. (2023). Positive affect, negative affect, and psychological resilience mediate the effect of self-compassion on mental toughness: a serial mediation analysis. Psychol. Sch. 60, 2991–3007. doi: 10.1002/pits.22902

[B29] Etherton K. Steele-Johnson D. Salvano K. Kovacs N. (2022). Resilience effects on student performance and well-being: the role of self-efficacy, self-set goals, and anxiety. J. Gen. Psychol. 149, 279–298. doi: 10.1080/00221309.2020.1835800 33111653

[B30] Feltz D. L. Lirgg C. D. (2001). “Self-efficacy beliefs of athletes, teams, and coaches,” in Handbook of Sport Psychology (New York, NY), 340–361.

[B31] Fletcher D. Sarkar M. (2013). “Psychological resilience,” in European Psychologist.

[B32] Fletcher D. Sarkar M. (2016). Mental fortitude training: an evidence-based approach to developing psychological resilience for sustained success. J. Sport Psychol. Action 7, 135–157. doi: 10.1080/21520704.2016.1255496

[B33] Fornell C. Larcker D. F. (1981). Structural Equation Models with Unobservable Variables and Measurement Error: Algebra and Statistics. Chicago, IL.

[B34] Galli N. Gonzalez S. P. (2015). Psychological resilience in sport: a review of the literature and implications for research and practice. Int. J. Sport Exerc. Psychol. 13, 243–257. doi: 10.1080/1612197X.2014.946947

[B35] Gross J. J. (2014). “Emotion regulation: conceptual and empirical foundations,” in Handbook of Emotion Regulation, 3–20.

[B36] Gross J. J. John O. P. (2003). Individual differences in two emotion regulation processes: implications for affect, relationships, and well-being. J. Pers. Soc. Psychol. 85:348. doi: 10.1037/0022-3514.85.2.348 12916575

[B37] Gucciardi D. F. (2017). Mental toughness: progress and prospects. Curr. Opini. Psychol. 16, 17–23. doi: 10.1016/j.copsyc.2017.03.010 28813344

[B38] Güngör S. (2021). The mediator role of the fear of covid-19 in the relationship between psychological resilience and life satisfaction. Curr. Psychol. 40, 6291–6299. doi: 10.1007/s12144-021-01525-w 33716474 PMC7937780

[B39] Hair J. F. Hult G. T. M. Ringle C. M. Sarstedt M. Danks N. P. Ray S. (2021). “An introduction to structural equation modeling,” in Partial Least Squares Structural Equation Modeling (PLS-SEM) Using R: A Workbook (Cham), 1–29.

[B40] Han J. Jin L. Yin H. (2024). Supervisors' emotion regulation in research supervision: navigating dilemmas in an accountability-based context. Higher Educ. 89, 671–689.

[B41] Hart R. (2024). Resilience Revolution. Oslo: Publifye AS.

[B42] Henseler J. Ringle C. M. Sarstedt M. (2015). A new criterion for assessing discriminant validity in variance-based structural equation modeling. J. Acad. Market. Sci. 43, 115–135. doi: 10.1007/s11747-014-0403-8

[B43] Hepler T. J. (2016). Can self-efficacy pave the way for successful decision-making in sport? J. Sport Behav. 39, 147–159.

[B44] Hess T. J. (2024). Effects of Imagery Skills Training on Self-Efficacy, Heart Rate, and Running Pace of Recreational Runners (PhD thesis). Capella University, Minneapolis, MN, United States.

[B45] Hu L. Bentler P. M. (1998). Fit indices in covariance structure modeling: Sensitivity to underparameterized model misspecification. Psychol. Methods 3:424. doi: 10.1037//1082-989X.3.4.424

[B46] Jannah M. Widohardhono R. Rachman N. Haba M. A. A. Harahap F. (2024). Athletes with disability: does emotional regulation affect mental toughness? Int. J. Disabil. Sports Health Sci. 7, 37–45. doi: 10.33438/ijdshs.1371348

[B47] Kashdan T. B. Weeks J. W. Savostyanova A. A. (2011). Whether, how, and when social anxiety shapes positive experiences and events: a self-regulatory framework and treatment implications. Clin. Psychol. Rev. 31, 786–799. doi: 10.1016/j.cpr.2011.03.012 21529701

[B48] Kay S. A. Merlo K. L. (2020). “Emotion regulation as a process to foster resilience,” in Research Handbook on Organizational Resilience (Cheltenham: Edward Elgar Publishing), 86–101.

[B49] Kim D. H. Kim J. H. Park K.-J. (2023). The impact of regular exercise, competition experience, and physical self-efficacy on psychological resilience. J. Sport Psychol. 32, 1–19.

[B50] Lazarus R. S. Folkman S. (1984). Stress, Appraisal, and Coping. Cham: Springer Publishing Company.

[B51] Lomax R. G. (2004). A Beginner's Guide to Structural Equation Modeling. New York, NY: Psychology Press.

[B52] Mahoney J. W. Ntoumanis N. Gucciardi D. F. Mallett C. J. Stebbings J. (2016). Implementing an autonomy-supportive intervention to develop mental toughness in adolescent rowers. J. Appl. Sport Psychol. 28, 199–215. doi: 10.1080/10413200.2015.1101030

[B53] Mendizabal B. (2024). The relationship between athletes' grit, mental toughness, and sport resilience. Phys. Educ. Stud. 28, 188–194. doi: 10.15561/20755279.2024.0401

[B54] Mutz J. Clough P. Papageorgiou K. A. (2017). Do individual differences in emotion regulation mediate the relationship between mental toughness and symptoms of depression? J. Individ. Differ. 38:a000224. doi: 10.1027/1614-0001/a000224

[B55] Nazarudin M. N. Singh S. S. B. Abdullah M. F. Pa W. A. M. W. (2025). Enhancing Youth Athletes' Self-Efficacy, Mental Skills, Emotional Management, and Rugby-Specific Skills Through the Super Rugby Program.

[B56] Nicholls A. R. Levy A. R. Carson F. Thompson M. A. Perry J. L. (2016). The applicability of self-regulation theories in sport: goal adjustment capacities, stress appraisals, coping, and well-being among athletes. Psychol. Sport Exerc. 27, 47–55. doi: 10.1016/j.psychsport.2016.07.011

[B57] Noble J. Vermillion M. Foster K. (2016). Coaching environments and student-athletes: Perceptions of support, climate and autonomy. Sport J. 19, 1–9.

[B58] Nüeztel C. (2023). Coping strategies for handling stress and providing mental health in elite athletes: a systematic review. Front. Sports Active Living 5:1265783. doi: 10.3389/fspor.2023.1265783 38033656 PMC10687549

[B59] Palamarchuk I. S. Vaillancourt T. (2021). Mental resilience and coping with stress: a comprehensive, multi-level model of cognitive processing, decision making, and behavior. Front. Behav. Neurosci. 15:719674. doi: 10.3389/fnbeh.2021.719674 34421556 PMC8377204

[B60] Podsakoff P. M. MacKenzie S. B. Lee J.-Y. Podsakoff N. P. (2003). Common method biases in behavioral research: a critical review of the literature and recommended remedies. J. Appl. Psychol. 88:879. doi: 10.1037/0021-9010.88.5.879 14516251

[B61] Reynolds A. J. McDonough M. H. (2015). Moderated and mediated effects of coach autonomy support, coach involvement, and psychological need satisfaction on motivation in youth soccer. Sport Psychol. 29, 51–61. doi: 10.1123/tsp.2014-0023

[B62] Rogowska A. M. Tataruch R. Niedzwiecki K. Wojciechowska-Maszkowska B. (2022). The mediating role of self-efficacy in the relationship between approach motivational system and sports success among elite speed skating athletes and physical education students. Int. J. Environ. Res. Public Health 19:2899. doi: 10.3390/ijerph19052899 35270591 PMC8910426

[B63] Sarkar M. Fletcher D. (2014). Psychological resilience in sport performers: a review of stressors and protective factors. J. Sports Sci. 32, 1419–1434. doi: 10.1080/02640414.2014.901551 24716648

[B64] Sha J. Tang T. Shu H. He K. Shen S. (2022). Emotional intelligence, emotional regulation strategies, and subjective well-being among university teachers: A moderated mediation analysis. Front. Psychol. 12:811260. doi: 10.3389/fpsyg.2021.811260 35082733 PMC8785825

[B65] Siebert A. (2009). The Resiliency Advantage: Master Change, Thrive Under Pressure, and Bounce Back from Setbacks (Victoria, BC).

[B66] Soflu H. G. Esfahani N. Assadi H. (2011). The comparison of emotional intelligence and psychological skills and their relationship with experience among individual and team athletes in superior league. Procedia-Soc. Behav. Sci. 30, 2394–2400. doi: 10.1016/j.sbspro.2011.10.466

[B67] Song B. Martínez-Aranda L. M. Leiva-Arcas A. Sánchez-Pato A. (2024). The evolution of chinese high-performance student-athletes' admission, cultivation and management policies. Int. J. Sport Policy Polit. 16, 151–175. doi: 10.1080/19406940.2023.2273350

[B68] Soundara Pandian P. Balaji Kumar V. Kannan M. Gurusamy G. Lakshmi B. (2023). Impact of mental toughness on athlete's performance and interventions to improve. J. Basic Clin. Physiol. Pharmacol. 34, 409–418. doi: 10.1515/jbcpp-2022-0129 35792085

[B69] Tokarska E. Rogowska A. M. (2025). Motivation and self-efficacy in cycling and running athletes: a person-centered approach. Front. Psychol. 16:1533763. doi: 10.3389/fpsyg.2025.1533763 40386667 PMC12083087

[B70] Usán Supervía P. Quílez Robres A. (2021). Emotional regulation and academic performance in the academic context: the mediating role of self-efficacy in secondary education students. Int. J. Environ. Res. Public Health 18:5715. doi: 10.3390/ijerph18115715 34073453 PMC8198487

[B71] van Rens F. E. Burgin M. Morris-Binelli K. (2021). Implementing a pressure inurement training program to optimize cognitive appraisal, emotion regulation, and sport self-confidence in a women's state cricket team. J. Appl. Sport Psychol. 33:402–419. doi: 10.1080/10413200.2019.1706664

[B72] Weinberg R. Butt J. Culp B. (2011). Coaches' views of mental toughness and how it is built. Int. J. Sport and Exerc. Psychol. 9, 156–172. doi: 10.1080/1612197X.2011.567106

[B73] Willis K. L. (2018). Hockey Grit, Grind & *Mind: Your Playbook for Increasing Toughness, Focus, Drive, Resilience, Confidence, and Consistency in Today's Game*. New York, NY: Morgan James Publishing.

[B74] Xin Z. Li T. Li Q. Chen Y. Wang M. (2024). Coach-athlete attachment and athlete burnout: testing longitudinal mediation via cognitive reappraisal and expressive suppression. Int. J. Sport Exerc. Psychol. 2024, 1–19. doi: 10.1080/1612197x.2024.2389207

[B75] Zagórska A. Guszkowska M. (2014). A program to support self-efficacy among athletes. Scand. J. Med. Sci. Sports 24, e121–e128. doi: 10.1111/sms.12125 24118561

[B76] Zhang N. Du G. Tao T. (2025). Empowering young athletes: the influence of autonomy-supportive coaching on resilience, optimism, and development. Front. Psychol. 15:1433171. doi: 10.3389/fpsyg.2024.1433171 39845556 PMC11750835

[B77] Zhou Y. Zhang Z. (2024). From athletic excellence to academic influence: a study of retired Chinese athletes transitioning into the higher education sector. Front. Psychol. 15:1401575. doi: 10.3389/fpsyg.2024.1401575 38957888 PMC11217518

